# *Campylobacter *spp. and bacteriophages from broiler chickens: Characterization of antibiotic susceptibility profiles and lytic bacteriophages

**DOI:** 10.1002/mbo3.784

**Published:** 2019-01-17

**Authors:** Anna Nowaczek, Renata Urban‐Chmiel, Marta Dec, Andrzej Puchalski, Dagmara Stępień‐Pyśniak, Agnieszka Marek, Ewelina Pyzik

**Affiliations:** ^1^ Sub‐Department of Veterinary Prevention and Avian Diseases, Faculty of Veterinary Medicine, Institute of Biological Basis of Animal Diseases University of Life Sciences Lublin Poland

**Keywords:** bacteriophage, *Campylobacter*, *Myoviridae*, phage therapy, poultry, *Siphoviridae*

## Abstract

Bacteria of the genus *Campylobacter* are the most common pathogens causing zoonotic diseases in humans. Therefore, the aim of the study was to isolate *Campylobacter* bacteria from broiler chickens and evaluate their susceptibility to selected antibiotics by determining minimum inhibitory concentrations (MIC), followed by isolation and characterization of bacteriophages specific for *Campylobacter *spp. The material for the study consisted of field isolates of *Campylobacter *spp. obtained from the gut (cecum) of broiler chickens directly after slaughter in slaughterhouses, and bacteriophages specific for these strains. We isolated 48 strains from poultry (140 broiler chickens): 31 strains of *Campylobacter jejuni* and 17 of *Campylobacter coli. *Identification of the strains was confirmed by multiplex PCR and MALDI‐TOF mass spectrometry. Over 83% of *Campylobacter* strains were resistant to ciprofloxacin, and over half the isolates were resistant to erythromycin, gentamicin, and tetracycline. Resistance to three or more antibiotics was observed in 91.6% of all strains. Four bacteriophages were obtained, and on the basis of their morphological structure, they were assigned to two families of the order *Caudovirales*: *Myoviridae* and *Siphoviridae*. A high percentage of the *Campylobacter* strains were resistant to at least three of the antibiotic groups tested. All of the phages exhibited lytic activity against the *Campylobacter* spp. isolates, but the antibacterial effect of the phages was not observed for all strains.

## 1 INTRODUCTION

Bacteria of the genus *Campylobacter *spp. mainly colonize the gut of birds past the age of 2–3 weeks. Despite their widespread occurrence in the intestines of birds, no visible disease symptoms are observed, and the significant economic losses are primarily due to the decrease in feed conversion and weight gains, and in the case of laying hens, reduced laying and hatching rates (Lemos, Morais, Fontes, Pires, & Vieira‐Pinto, 2015). Despite the low virulence of the bacteria for birds, certain strains can induce severe food poisoning in humans, posing a significant health problem worldwide. According to the European Food Safety Authority (EFSA), this is one of the most commonly reported zoonoses in the European Union, and the number of confirmed cases of infection has shown an upward trend in recent years. According to reports published by the European Food Safety Authority (EFSA), campylobacteriosis is one of the most frequently reported zoonotic diseases in humans in the European Union, with 246,307 confirmed cases in 2016, which was 10.5% higher than in the previous year (EFSA & ECDC, 2014, 2015, 2016). Widespread drug resistance in bacteria and legal restrictions on the use of antibiotics in livestock farming, particularly in the EU, and since 2009 in the United States and Canada as well, have led to the need for new solutions to eliminate pathogenic (and in particular zoonotic) bacteria, in order to ensure the safety of raw materials used for food (Maron, Smith, & Nachman, 2013). According to the EU/EEA, the prevalence of multiresistant *Campylobacter *spp. isolates in EU countries in 2012 was over 28% (EFSA and ECDC, 2014).

One possible solution providing an alternative to antibiotics is the use of bacteriophages, a group of viruses infecting bacterial cells. Phages lack the cell structure and enzyme systems essential for food intake and protein synthesis and can replicate only in living cells (Hagens & Loessner, 2010). This is the largest group of viruses, surpassing the number of bacteria ten times (10^31^). They are present throughout the environment, for example, in water and wastewater, soil, human and animal feces, and products of plant (fruits and vegetables) and animal origin (Andreatti Filho et al., 2007; O'Flaherty, Ross, & Coffey, 2009). Mainly on the basis of genome type and virion morphology, bacteriophages were assigned to 873 species, 204 genera, and 14 subfamilies in the 2015 taxonomy release ICTV report (International Committee on Taxonomy of Viruses, EC 48, Budapest, Hungary, August 2016). However, the vast majority (about 96%) belong to the families *Myoviridae*, *Podoviridae,* or *Siphoviridae*, which are phylogenetically related and comprise the order *Caudovirales* (Weinbauer, 2004; Wernicki, Nowaczek, & Urban‐Chmiel, 2017).

The antibacterial properties of phages have found application in experimental therapies in humans and animals and in the development of disinfectants eliminating bacteria from the surfaces of foods of plant and animal origin (Abuladze et al., 2008; Carlton, Noordman, Biswas, Meester, & Loessner, 2005; Lim et al., 2011; Weber‐Dąbrowska, Mulczyk, & Górski, 2000).

The aim of the study was to isolate *Campylobacter *spp. strains from broiler chickens and to evaluate their susceptibility to selected antibiotics by determining minimum inhibitory concentrations (MIC), and then to isolate and characterize bacteriophages specific for *Campylobacter *spp.

## 2 MATERIALS AND METHODS

### 2.1 Isolation and morphological analysis of *Campylobacter* spp.

The material for the study consisted of field isolates of *Campylobacter* spp. obtained from the gut (cecum) of 140 broiler chickens directly after slaughter in slaughterhouses in southeastern Poland in September and October. The birds were from different indoor flocks. Presumptive identification of *Campylobacter *spp. isolates was based on colony morphology, Gram staining, and growth in microaerobic conditions. Initial isolation was carried out in Bolton Broth (Oxoid Ltd., UK). The cultures were incubated at 37°C for 48 hr in microaerophilic conditions (5% O_2_, 10% CO_2_, 85% N) in the CampyGen system (Oxoid Ltd.). On media that showed growth of gray, flat, and moist bacterial colonies with a tendency to expand, single colonies belonging morphologically to the *Campylobacter* spp. type were collected, directly transferred to selective mCCDA agar, and incubated at 41.5°C for 48 hr in microaerophilic conditions (Dudzic et al., 2016). The isolates were stored at −80°C in the Microbank system for storage of micro‐organisms (Biocorp, PL).

### 2.2 Genetic identification of bacteria by multiplex PCR and MALDI‐TOF mass spectrometry

DNA was isolated using a commercial DNA purification kit (GeneMatrix Bacterial & Yeast Genomic DNA Purification Kit; EURx, PL) according to the manufacturer’s instructions. The genus and species identification of the bacterial isolates was confirmed by multiplex PCR using specific primers according to own previous study (Dudzic et al., 2016). PCR was carried out using primers amplifying the 16S rRNA gene of *Campylobacter jejuni* and *Campylobacter coli* to determine genus (*MD16S1* and *MD16S2l*), as well as primers amplifying the *mapA* gene of *C. jejuni* (*MDmapA1* and *MDmapA2*) and the *ceuE* gene of *C. coli* (*MDCOL3* and *MDCOL2*). Reference strains of *C. jejuni *NCTC 12662 and *C. coli *ATCC 33559 were used as positive controls. The primer sequences and conditions are given in Table 1.

**Table 1:** Sequences of primers and conditions specific for *Campylobacter *spp. identification in multiplex PCR

Primer	Primer sequence (5′→3′)	Name of gene	Melting temperature (°C)	Size of amplicon (bp)
*MD16S1*	ATCTAATGGCTTAACCATTAAC	*16SrRNA* *Campylobacter jejuni* *+* *Campylobacter Coli*	51.0	857
*MD16S2l*	GGACGGTAACTAGTTTAGTAT		50.5	
*MDmapA1*	CTATTTTATTTTTGAGTGCTTGTG	*mapA* *C. jejuni*	50.7	589
*MDmapA2*	GCTTTATTTGCCATTTGTTTTATA		51,2	
*MDCOL3*	AATTGAAAATTGCTCCAACTATG	*ceuE* *C. coli*	51.7	462
*MDCOL2*	TGATTTTATTATTTGTAGCAGCG		51.3	

Amplification reactions were carried out in a thermal cycler (Eppendorf Mastercycler gradient, USA) using the following program: One cycle at 94°C/5 min, 30 cycles 58°C/1 min, 72°C/1 min, 94°C/1 min, and one cycle 72°C/5 min. The amplification products were evaluated in a 1.5% agarose gel at 100 V. A 100–1,000 bp molecular weight standard (Blirt, PL) was used to determine the size of the amplification products. The electrophoresis images were analyzed in UV light using Gel‐Doc 2000 (Bio‐Rad, USA).

Species identification of the isolates was additionally confirmed by MALDI‐TOF mass spectrometry (UltrafleXtreme MALDI‐TOF [Bruker Daltonics, Germany]). We analyzed single 24 hr colonies grown on Columbia medium with 5% defibrinated sheep blood following protein extraction with ethanol and formic acid (Sigma‐Aldrich, Poland). For this purpose, 900 µl of absolute ethanol was added to bacteria suspended in 300 µl deionized water and mixed thoroughly on a vortex mixer for 1 min. The samples were centrifuged (13,000 *g*/2 min/20°C), the supernatant was removed, and 50 µl each of 70% formic acid and acetonitrile was added to the precipitate. Next the samples were mixed by repeated pipetting and on a vortex mixer. After centrifuging again at 13,000 *g*/2 min/20°C, 1 µl of the supernatant was applied to a metal plate (MTP, AnchorChip™ T F stainless steel MALDI target plate; Bruker), which had previously been activated with trichloroacetic acid and left to dry for 10 min at room temperature. Then, the plate with the bacterial suspension was covered with 1 µl of matrix solution and analyzed in an UltrafleXtreme MALDI‐TOF mass spectrometer (Bruker Daltonics).

### 2.3 Antibiotic susceptibility of *Campylobacter* spp. isolates

The susceptibility of the isolated *Campylobacter* strains to selected antibiotics was tested by determining the minimum inhibitory concentrations (MIC) in Mueller Hinton broth (Sigma‐Aldrich). MICs were determined by the broth microdilution procedure in 96‐well flat‐bottomed microtiter plates according to Andrews (2001). The antibiotics tested were ciprofloxacin (CIP; Fluka), amoxicillin (AML; Fluka), ampicillin (AMP; Roth, Poland), gentamicin (CN; Roth), streptomycin (S; Roth), tetracycline (TE; Roth), erythromycin (E; Roth), and chloramphenicol (C; Roth). The concentrations for the antibiotics ranged from 0.5 to 256 µg/ml for tetracycline and erythromycin; from 0.25 to 128 µg/ml for amoxicillin, ampicillin, chloramphenicol, erythromycin, and gentamicin; and from 0.125 to 64 µg/ml for ciprofloxacin. The plates were incubated for 24 hr at 42°C in microaerophilic conditions. A *C. jejuni* reference strain (ATCC 33560) was used as a control. All tests were run in triplicate. The *Campylobacter* spp. isolates were classified as resistant or susceptible according to EUCAST, European Committee on Antimicrobial Susceptibility Testing 2016a, 2016b.

### 2.4 Isolation of bacteriophages specific for *Campylobacter* spp.

Bacteriophages were isolated from 70 fecal samples collected from 20 different farms. To isolate bacteriophages, we used 10 g of chicken feces suspended in 100 ml SM buffer with 2% gelatine. The 10% *w*/*v* suspension obtained in this manner was incubated in a rocking shaker at 4°C/120 rpm overnight. Then, the supernatant was centrifuged at 13,000 *g*/10 min and filtered through syringe filters 0.45 and 0.22 µm in diameter (Roth). The presence of bacteriophages in the lysate was confirmed by the double‐layer plate method according to Loc Carrillo, Connerton, Pearson, and Connerton (2007). For this purpose, 200 µl of bacterial suspension was added to 4 ml of NZCYM broth supplemented with 0.7% agar at about 45°C with 1 M CaCl_2 _and 1 M MgSO_4_. This was mixed thoroughly and poured onto a plate with NZCYM medium supplemented with 1.2% agar. The phage lysate was applied to the surface of the plate in the form of a 10 µl drop of cooled top agar. After 20 min of incubation at room temperature, the plates were transferred to an incubator and incubated at 42°C for 24 or 48 hr in microaerophilic conditions. The presence of bacteriophages was confirmed by observation of zones of inhibition of bacterial growth (lysis). Before further characterization, the phages were individually plaque‐purified three times on agar plates according to Han et al. (2013).

To determine the lytic activity spectrum of the bacteriophages, 10 µl of bacteriophage suspension was placed on plates containing individual strains of *Campylobacter* spp. and incubated for 48 hr in microaerophilic conditions. The bacteriophage activity spectrum was determined on the basis of zones of inhibition of bacterial growth in the form of plaques, as the lytic effect of the phage activity. The bacteriophages were evaluated for their lytic activity against all *Campylobacter *strains isolated from poultry during the study.

Two bacteriophages specific for *C. jejuni* (φ198 and φ287), obtained from Prof. Lone Brøndsted (University of Copenhagen, Denmark), were additionally used as reference phages for comparison with the wild‐type phages obtained in this study.

### 2.5 Determination of the titer of bacteriophages specific for *Campylobacter* spp.

The phage titer was determined by serial dilutions of the phage lysate suspended in SM buffer. Titration of each phage was carried out using a *Campylobacter* strain. A 10 µl volume of phage lysate was added to Eppendorf tubes containing 90 µl of TM buffer and then vortexed. Then, 10 µl of the suspension was transferred to new tubes containing 90 µl of TM buffer. The process was repeated until a 10^−8^ dilution was attained. A 2.5 µl volume of each of the phage dilutions was placed on the plate with 1.5% NZCYM medium and prepoured agar top agar containing *Campylobacter*. The plates were left at room temperature for 20 min and then incubated overnight at 42°C in microaerophilic conditions. The phage titer was determined on the basis of the presence of lysis zones (plaques) in each dilution and was the last dilution of the phage suspension at which plaques were observed.

### 2.6 Morphological analysis of bacteriophages specific for *Campylobacter *spp. by transmission electron microscopy

Morphological analysis of the bacteriophages was carried out by transmission electron microscopy of negatively stained slides. For this purpose, 5 ml of each phage suspension in TM buffer was adsorbed onto glow‐discharged carbon‐coated 200 mesh copper grids, and then, after 3 min adsorption, the excess lysate was collected and the slides were negatively stained with 2% uranyl acetate (pH 4.0) for 15 s. The slides were examined using a Philips CM 100 transmission electron microscope at 12,000–80,000× magnification, and selected phages were recorded using iTEM software (Olympus Soft Imaging Solution). The bacteriophages were classified according to Carvalho, Gannon, et al. (2010), Carvalho, Susano, et al. (2010) and Furuta et al. (2017).

### 2.7 DNA restriction analysis of phages

DNA obtained from the *Campylobacter* bacteriophages was subjected to double digestion with the restriction enzymes *Hind*III + ClaI and* Mse*I + TaqI (Thermo Scientific, USA). Samples prepared according to the manufacturer’s instructions were incubated at 37°C (for enzymes *Hind*III and *Cla*I) and 65°C (for* Mse*I and *Taq*I) for 4 hr. Restriction analysis was carried out in a 2% agarose gel in TBE buffer (1 M Tris‐HCl pH 8.0; 10 ml 0.5 M EDTA [pH 8.0], distilled H_2_O) at 70 V for 50–60 min. The electropherograms were analyzed using Gel‐Doc 2000 in UV light (Bio‐Rad). A 100–3,000 bp molecular weight standard (Blirt) was used to determine the size of the restriction products.

## 3 RESULTS

**Figure 1:**
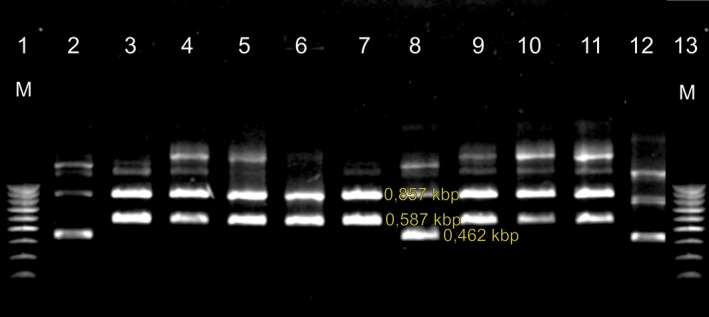
PCR analysis of DNA obtained from *Campylobacter jejuni* and *Campylobacter coli*. Legend: Lanes 1 and 13—100–1,000 bp molecular weight marker (MR61; Blirt S.A); lane 2—*C. jejuni* reference strain (NCTC 12,662)—16S rRNA gene amplicons—0.857 kbp and *mapA*—0.587 kbp; 3—*C. coli* reference strain (ATCC 43,479)—gene amplicons 16S rRNA‐ 0,857 kbp and *ceuE*—0,462 kbp; lanes 4–7 and 9–11 *C. coli* wild‐type strains; lanes 8, 12—*C. jejuni* wild‐type

We obtained 48 bacterial strains belonging to the genus *Campylobacter *spp.; species identification in MALDI‐TOF mass spectrometry was confirmed for the 48 isolates, among which 31 strains were classified as *C. jejuni* and 17 as *C. coli*. For 34 strains, the identification score ranged from 2.0 to 2.299, which indicated reliable identification to the genus level and probable identification to the species level. For 11 strains, the identification score ranged from 1.7 to 1.99, and three strains had a score below 1.700 (Table 2). Analysis of the electrophoresis profiles of the PCR products confirmed the presence of the 16S rRNA gene specific for *Campylobacter *spp. in all 48 isolates. Species differentiation identified 31 isolates of the species *C. jejuni* based on the gene *mapA* and 17 isolates of *C. coli* based on the gene *ceuE* (Figure 1). The isolates were from five different farms located in southeastern Poland.

**Table 2:** The mean log (score) results of MALDI‐TOF MS analysis for *Campylobacter *spp. isolated from poultry

Log (score)	Description	Symbol	Number of test strains	Total percentage of strains (%)
2.000–2.299	Secure genus identification and species identification	+++	34	70.8
1.700–1.999	Genus identification and medium species identification	++	11	22.9
<1.700	Genus identification and low species identification	+	3	6.25

Analysis of drug susceptibility based on the minimum inhibitory concentration for each of the antibiotics showed that the highest percentages of *C. jejuni* strains were resistant to ciprofloxacin (CIP)—80.6% and tetracycline (TE)—64.5%. More than half of the isolates were resistant to erythromycin (E) and gentamicin (CN)—51.6%. The percentage of strains resistant to streptomycin was 35.4%. In the case of β‐lactam antibiotics, the level of resistance to ampicillin (AMP) was 38.7% and to amoxicillin (AML) 29%. The lowest resistance among the *C. jejuni* strains was observed for chloramphenicol (C)—(12.9%). In the case of *C. coli*, as for *C. jejuni*, the highest percentages of strains were resistant to tetracycline (94.1%) and ciprofloxacin (88.2%). High resistance among *C. coli* isolates was noted for gentamicin (64.7%), erythromycin (64.7%), and ampicillin (58.8%). Nearly half of the bacteria were resistant to amoxicillin (47%), and about 35% to chloramphenicol. These strains showed the lowest resistance to streptomycin—17.6% (Tables 3 and 4). The antibiotic profiles of the *Campylobacter *strains are presented in the Supporting Information Table S3a.

**Table 3:** Minimal inhibitory concentrations of antibiotics for *Campylobacter jejuni* and *Campylobacter coli*

Antibiotic	Isolated strains	Number of strains with given MIC (µg/ml) at given concentration of antibiotic											Number of resistant strains/total strains
		≥256	128	64	32	16	8	4	2	1	0.5	≤0.25	
AML	*C. jejuni*	8		1		1		1			8	12	9/31
	*C. coli*	5			2	1			1			8	8/17
AMP	*C. jejuni*	8	1		2	1	1			6	6	6	12/31
	*C. coli*	5		3	2			1			1	5	10/17
CIP	*C. jejuni*	2		1	2	1	3	5	5	4	2	6	25/31
	*C. coli*	2		2		1	1	1	5	1	2	2	15/17
CN	*C. jejuni*	4		1	6	4		1	3		2	10	16/31
	*C. coli*	1		3	1	3	2	1	1		2	3	11/17
TE	*C. jejuni*	7		2	5	5		1		2	1	8	20/31
	*C. coli*	6		1	3	4	2				1		16/17
C	*C. jejuni*	3		1		2	6	8	5	1		5	4/31
	*C. coli*	4		2			1	4	3	1		2	6/17
E	*C. jejuni*	15		1					7	1	4	3	16/31
	*C. coli*	11						2	1			3	11/17
S	*C. jejuni*				2		9	2	6	5	3	4	11/31
	*C. coli*					2	1	4	2	2	4	2	3/17

Note

AML: amoxicillin; AMP: ampicillin; C: chloramphenicol; CIP: ciprofloxacin; CN: gentamicin; E: erythromycin; S: streptomycin; TE: tetracycline.

**Table 4:** Number of drug‐resistant strains of *Campylobacter *spp. determined on the basis of MIC threshold values

Number of strains resistant to	*Campylobacter jejuni* *n* = 31 (%)	*Campylobacter coli* *n* = 17 (%)	Total (%)
At least one substance	31 (100)	17 (100)	48 (100)
At least three groups of antibiotics	27 (87)	17 (100)	44 (91.6)
AML	9 (29)	8 (47)	17 (35.41)
AMP	12 (38.7)	10 (58.8)	22 (45.8)
CIP	25 (80.6)	15 (88.2)	40 (83.3)
CN	16 (51.6)	11 (64.7)	27 (56.2)
TE	20 (64.5)	16 (94.1)	36 (54.1)
C	4 (12.9)	6 (35.2)	10 (20.8)
E	16 (51.6)	11 (64.7)	27 (56.2)
S	11 (35.4)	3 (17.6)	14 (29.1)

Note

AML: amoxicillin; AMP: ampicillin; C: chloramphenicol; CIP: ciprofloxacin; CN: gentamicin; E: erythromycin; S: streptomycin; TE: tetracycline.

In the present study, we isolated four bacteriophages, which showed lytic properties against 12 of the 48 *Campylobacter* spp. test strains. The lytic activity of the phages was determined on the basis of the presence of clear zones on the double‐layer plates. The appearance of a clear zone after 24 or 48 hr of incubation in microaerophilic conditions was interpreted as the presence of phage particles. The antibacterial activity of the phages was observed against 17 *Campylobacter* strains. The lytic titers of the phages were determined in triplicate for four wild‐type *Campylobacter *strains common to all tested phages and one reference strain of *C. jejuni *(NCTC 12662). The bacteriophage titers ranged from 1.9 × 10^2^ to 2.0 × 10^8^ PFU/ml. The highest titer was obtained in the case of phage φ297 (2.0 × 10^8^ PFU/ml), and the lowest for bacteriophage φ4 (1.9 × 10^1^ PFU/ml). Lytic activity of φ22 was confirmed in the case of four strains of *C. jejuni*. Activity of φ44 was confirmed in the case of three isolates of *C. jejuni* and one strain of *C. coli.* Phage φCj1 exhibited lysis against eight strains of *C. jejuni*. Bacteriophages φ198 and φ287 exhibited lytic activity against 12 *Campylobacter* strains (φ198 against eight isolates of *C. jejuni* and φ4 against seven isolates of *C. jejuni*; Table 5).

**Table 5:** Types, titers and lytic activity spectrum of bacteriophages specific for *Campylobacter jejuni* and *Campylobacter coli* strains

Phage no.	Family	Head (nm)/Tail (nm)	No. of host *Campylobacter* strain	Lytic titer PFU/ml	Lytic activity spectrum^a^
φ4	*Myoviridae*	100/99	Cj4	1.9 × 10^−2^	6
φ44	*Myoviridae*	100/100	Cj44	1.4 × 10^−2^	4
φ22	*Siphoviridae*	80/180	Cj22	2.4 × 10^−2^	4
φCj1	*Siphoviridae*	68/157	Cj1	1.9 × 10^−3^	3
φ198	*Myoviridae*	100/120	NCTC 12662	2.0 × 10^−8^	8
φ287	*Myoviridae*	100/120	NCTC 12662	1.3 × 10^−8^	7

a Number of *Campylobacter* spp. strains lysed by phages.

**Figure 2:**
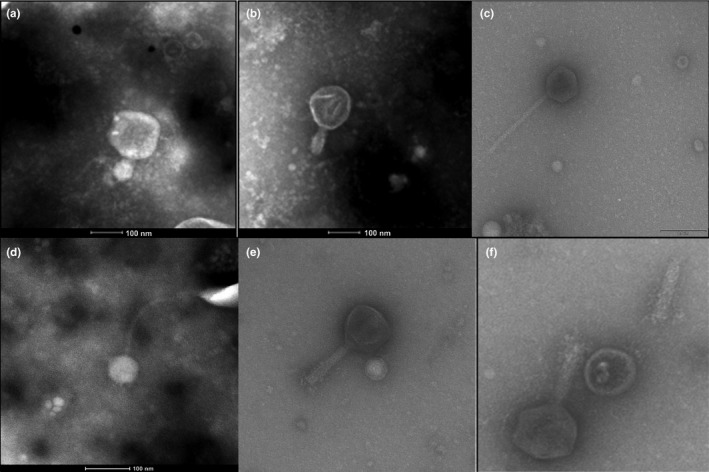
Morphological image of negative‐stained electron micrographs of bacteriophages specific for *Campylobacter* spp. Legend: *Myoviridae*‐like phages; (a)—wild‐type φ4; (b)—wild‐type φ44 (pentagonal head type); (e)—reference φ198; (f)—reference φ287 (Normal pentagonal heads with extender tail); *Siphoviridae*‐like phages; (c)—wild‐type φ22; (d)—wild‐type φCj1 (Small pentagonal heads with long thin tails)

Morphological analysis of the bacteriophages revealed the presence of virions specific for *Campylobacter *spp. with a structure typical of the order *Caudovirales*. Four of the bacteriophages were assigned to the family *Myoviridae *and two to the family *Siphoviridae*. The bacteriophages assigned to the family *Myoviridae *had elongated icosahedral heads (100–120 nm) without an envelope, and contractile tails with visible sheaths and an average length of 100 nm. The two bacteriophages assigned to the family *Siphoviridae* consisted of a symmetrical icosahedral head 68 and 80 nm in diameter and a tail 157 and 180 nm long (Figure 2). The phages exhibited lytic activity against 16 strains of *C. jejuni*. There were 32 isolates resistant to all phages.

**Figure 3:**
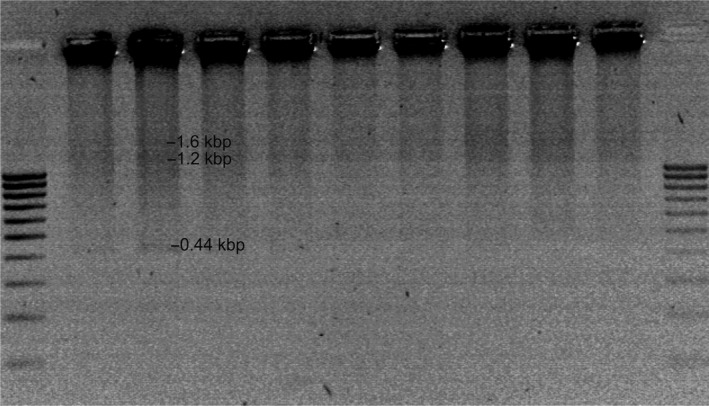
Restriction analysis of the genome of bacteriophages specific for *Campylobacter *spp. strains isolated from poultry after double digestion with the enzymes *Hind*III and *Cla*I. Legend: Lanes 1 and 11—100–1,000 bp molecular weight marker (MR61; Blirt S.A); lane 2—reference φ198, lane 3—reference φ289, lane 4—wild‐type phages φCj1, φ4, φ22, lanes 7–10—wild‐type phage φ44

**Figure 4:**
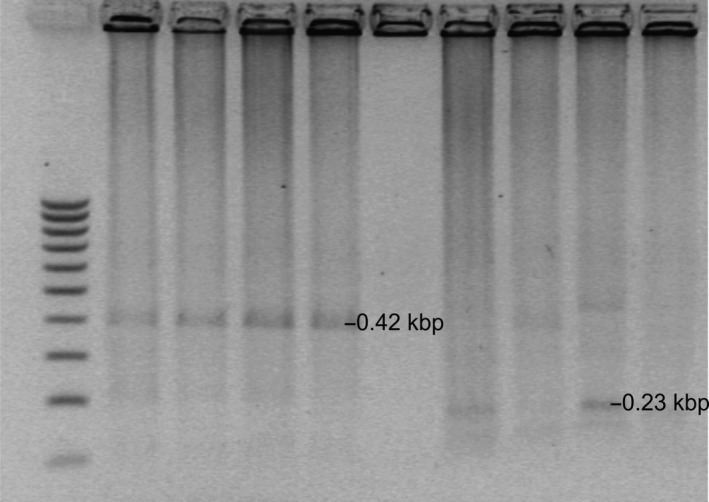
Restriction analysis of the genome of bacteriophages specific for *Campylobacter *spp. strains isolated from poultry after double digestion with *Mse*I and *Tag*I. Legend: Lane 1—100–1,000 bp molecular weight marker (MR61; Blirt S.A); lane 2—reference φ198, lane 3—reference φ289, lanes 4, 5—wild‐type phages φCj1, φ4, lanes 7, 8—wild‐type phages φ22, lanes 9/10 wild‐type phage φ44

Restriction analysis of phage material in double digestion with *Hind*III + ClaI and *Mse*I + TaqI revealed the presence of single restriction fragments. In the case of digestion with *Hind*III and *Cla*I, two main fragments were obtained, of 1.6 and 1.2 kbp, in all profiles analyzed. For the reference phages 198 and 287, a 0.44‐kbp fragment was observed as well (Figure 3). Double digestion with *Mse*I and *Taq*I produced a fragment of about 0.42 kbp, which was present in all profiles, and a fragment of 0.23 kbp visible in the profile of wild‐type phages 22 and 44. For φ44, there was also a small restriction fragment of 0.47 kbp (Figure 4). The rest of the material was not digested, as shown in the Figures, and thus, a comprehensive analysis of the phage profiles was not possible.

## 4 DISCUSSION

Bacteria of the genus *Campylobacter *spp. appear in a few chicks at the end of the first week after hatching, and then spread very quickly through the entire flock between the ages of 2 and 3 weeks. *Campylobacte*r bacteria are one of the main causes of contamination of poultry carcasses in the slaughterhouse, and these carcasses are the primary source of campylobacteriosis infections in humans (Hwang et al., 2009).

In our study, morphological analysis, PCR techniques, and MALDI‐TOF mass spectrometry were used to isolate 48 strains of *Campylobacter *spp. and assign them to two species: *C. jejuni* and *C. coli*. The positive results of identification using MALDI‐TOF mass spectrometry, as well as results obtained by other authors, confirm that these diagnostic techniques can be used to identify this type of pathogen (Bessede, Solecki, Sifré, Labadi, & Mégraud, 2011; Martiny, Visscher, Catry, Chatellier, & Vandenberg, 2013) and other microbial isolates from birds (Dec, Nowaczek, Urban‐Chmiel, Stępień‐Pyśniak, & Wernicki, 2018; Stępień‐Pyśniak, Hauschild, Różański, & Marek, 2017).

The strains were isolated from most of the samples collected from broiler chickens intended for slaughter. Of 70 fecal samples collected from the chickens, 48 tested positive, which was 68.5% of all samples tested. High carriage of this genus of bacteria in birds is confirmed by the latest research conducted by a team from Thailand (Prachantasena et al., 2016), who reported the occurrence of these bacteria in 75% of birds tested. Results obtained by British researchers showed the presence of *Campylobacter* spp. in 42.3% of all samples collected from birds (Bardon, Kolar, Cekanova, Hejnar, & Koukalova, 2009; Coles, McCarthy, Bliss, Layton, & Maiden, 2015; Ingresa‐Capaccioni et al., 2015). The present study found higher prevalence of *C. jejuni* than of *C. coli*, which was confirmed by PCR and MALDI‐TOF analysis.

Our study showed that the isolated *Campylobacter *spp. bacteria had high resistance to antibiotics commonly used in poultry production to control infections. This is confirmed by the minimum inhibitory concentrations and is consistent with studies carried out by international research centers. A high percentage of resistant strains was observed in the case of ciprofloxacin, at a level of 80.6% for *C. jejuni *and 88.2% for *C. coli*. These results are consistent with those obtained by other authors (EFSA & ECDC, 2016; Wieczorek, Szewczyk, & Osek, 2012), which have confirmed a high rate of resistance to this antibiotic, ranging from 69.8% to 88.1% of strains.

In the case of other antibiotics, our results demonstrated a high rate of resistance to erythromycin (*C. jejuni*—51.6%, *C. coli*—64.7%), which differed somewhat from results presented by other authors, who have found no more than 2.5% of strains resistant to erythromycin (Wieczorek & Osek, 2015; Woźniak & Wieliczko, 2011). One possible cause of such a high percentage of resistance to erythromycin could be unacknowledged use of this antibiotic in the flocks tested. On the other hand, the high percentage of *Campylobacter* spp. isolates resistant to tetracycline (*C. jejuni*—64.5%, *C. coli*—94.1%) in our study is supported by other authors, who have reported resistance to tetracycline in over 55% of all strains (Wieczorek & Osek, 2015; Woźniak & Wieliczko, 2011).

The growing phenomenon of bacterial resistance to commonly used antibiotics in both human and veterinary medicine necessitates the search for alternative methods to combat bacterial pathogens. Phages specific for *Campylobacter* spp. are used to reduce the occurrence of this pathogen in the gut of poultry, in order to limit economic losses resulting from reduced productivity, weight gains, and feed conversion, as well as to reduce the risk of contamination of meat for human consumption (Carvalho, Gannon, et al., 2010; Loc Carrillo et al., 2005). Bacteriophages exhibiting specificity for *Campylobacter* spp. can be used successfully to eliminate bacteria from carcass surfaces (Atterbury, Connerton, Dodd, Rees, & Connerton, 2003).

In the present study, we obtained four bacteriophages exhibiting specificity for 17 strains of *Campylobacter* spp. This was presumably linked to the fact that the samples were obtained from indoor poultry housing systems, which probably had a significant influence on the number of bacteriophages obtained for the tested *Campylobacter *strains.

A similar low percentage of phages specific for *Campylobacter *spp. was observed in a study by Hansen, Rosenquist, Baggesen, Brown, and Christensen (2007), in which only five bacteriophages were obtained for 222 fecal samples from broiler chickens. In a study by Atterbury, Connerton, Christine, et al. (2003), phages were obtained from only 11% of all samples used for isolation of bacterial viruses. Firlieyanti, Connerton, and Connerton (2016) have also reported a low 2.7% isolation frequency of *Campylobacter‐*specific bacteriophages. According to other authors (Carvalho, Susano, et al., 2010; Owens, Barton, & Heuzenroeder, 2013), the low activity of bacteriophages against *Campylobacter* spp. may be due to the origin of these bacteria. These authors suggest that the most phage‐positive samples are usually obtained from birds kept in backyard farming systems rather than closed systems.

Most bacteriophages exhibiting specificity for *Campylobacter* bacteria have a structure typical of phages of the family *Myoviridae *(Hansen et al., 2007; Hwang et al., 2009; Sorensen et al., 2015). In the present study, however, in addition to the phages assigned to the *Myoviridae* family, we also obtained phages which were assigned to the *Siphoviridae* family on the basis of their morphological structure. This is interesting because the available literature concerning strains of *Campylobacter *spp. includes few reports describing bacteriophages of the family *Siphoviridae *(Ackermann, 2007; Sails, Wareing, Bolton, Fox, & Curry, 1998).

Restriction analysis of the phage material obtained using double digestion with *Hind*III + ClaI and *Mse*I* *+ TaqI confirmed the presence of single restriction fragments, which was insufficient for a comprehensive comparative analysis of the phages. The fragments obtained in both cases confirmed that the bacteriophage profiles were highly similar, as indicated by common fragments of 1.6 and 1.2 kbp in all profiles following digestion with *Hind*III and *Cla*I and the 0.42‐kbp fragment obtained by double digestion with *Mse*I and *Taq*I. Similar difficulties with restriction analysis have been reported by Loc Carrillo et al. (2007), who used 12 restriction enzymes and achieved digestion of the genetic material for four only different phages (CP 2, 4, 5 and 8) specific for *Campylobacter *spp. The similar restriction profiles obtained consisted of four main fragments ranging from 4 to 64 kbp. In another study (Hansen et al., 2007), analysis of genomic DNA obtained from 34 phages revealed variation in the restriction profiles of five phages, while in the case of one phage, no digestion effect was obtained. In a study by Atterbury, Connerton, Christine, et al. (2003) and Atterbury, Connerton, Dodd, et al. (2003) a restriction effect was visible in the case of six of 12 bacteriophages.

## 5 CONCLUSION

To sum up, the results of the study indicate that bacteria of the genus *Campylobacter* spp. are characterized by a high frequency of strains resistant to at least three of the antibiotic groups tested. The occurrence of bacteriophages specific for *Campylobacter* spp. strains is very low; the use of material from the living environments of birds raised in backyard systems is much more conducive to the acquisition of bacteriophages. Restriction analysis of phages requires testing of many enzymes using the double digestion method, which makes it possible to obtain more restriction fragments. A full genetic characterization of the phages obtained in the present study will be performed as the next stage of our research and will involve the use of more bacteriophages obtained from poultry environments.

## CONFLICT OF INTEREST

None declared.

## AUTHOR CONTRIBUTION

AN performed the experiments, analyzed data, and contributed to drafting the manuscript. RU‐C conceived the study, participated in the preparation of the manuscript, analyzed the results, and contributed to drafting the manuscript. MD participated in the laboratory part of the study involving restriction analysis. AP participated in isolation and characterization of the bacteria and bacteriophages, performed the experiments, and analyzed the data. DS‐P participated in bacteriophage isolation and cultures of *Campylobacter* spp. strains. AM participated in bacteriophage isolation. EP participated in bacteriophage isolation. All authors have approved the final version of the manuscript.

## ETHICS STATEMENT

Ethical clearance was not applicable to this study as no animals were used.

## Data Availability

All data are provided in full in the Results section of this paper.

## References

[mbo3784-bib-0001] Abuladze, T. , Li, M. , Menetrez, M. Y. , Dean, T. , Senecal, A. , & Sulakvelidze, A. (2008). Bacteriophages reduce experimental contamination of hard surfaces, tomato, spinach, broccoli, and ground beef by *Escherichia coli *O157:H7. Applied Environmental Microbiology, 74, 6230–6238. 10.1128/AEM.01465-08 18723643 PMC2570303

[mbo3784-bib-0002] Ackermann, H. W. (2007). 5500 phages examined in the electron microscope. Archives of Virology, 152, 227–243. 10.1007/s00705-006-0849-1 17051420

[mbo3784-bib-0003] Andreatti Filho, R. L. , Higgins, J. P. , Higgins, S. E. , Gaona, G. , Wolfenden, A. D. , Tellez, G. , & Hargis, B. M. (2007). Ability of bacteriophages isolated from different sources to reduce *Salmonella enterica *serovar enteritidis in vitro and in vivo. Poultry Sciences, 86, 1904–1909. 10.1093/ps/86.9.1904 17704377

[mbo3784-bib-0004] Andrews, J. M. (2001). Determination of minimum inhibitory concentrations. Journal of Antimicrobial Chemotherapy, 48, 5–16. 10.1093/jac/48.suppl_1.5 11420333

[mbo3784-bib-0005] Atterbury, R. J. , Connerton, P. L. , Christine, E. R. , Dodd, C. E. R. , Rees, C. E. D. , & Connerton, I. F. (2003). Isolation and characterization of *Campylobacter *bacteriophages from retail poultry. Applied Environmental Microbiology, 69, 4511–4518. 12902236 10.1128/AEM.69.8.4511-4518.2003 PMC169066

[mbo3784-bib-0006] Atterbury, R. J. , Connerton, P. L. , Dodd, C. E. , Rees, C. E. , & Connerton, I. F. (2003). Application of host‐specific bacteriophages to the surface of chicken skin leads to a reduction in recovery of *Campylobacter jejuni* . Applied Environmental Microbiology, 69, 6302–6306. 14532096 10.1128/AEM.69.10.6302-6306.2003 PMC201188

[mbo3784-bib-0007] Bardon, J. , Kolar, M. , Cekanova, L. , Hejnar, P. , & Koukalova, D. (2009). Prevalence of *Campylobacter jejuni* and its resistance to antibiotics in poultry in the Czech Republic. Zoonoses Public Health, 56, 111–116. 18771516 10.1111/j.1863-2378.2008.01176.x

[mbo3784-bib-0008] Bessède, E. , Solecki, O. , Sifré, E. , Labadi, L. , & Mégraud, F. (2011). Identification of Campylobacter species and related organisms by matrix assisted laser desorption ionization‐time of flight (MALDI‐TOF) mass spectrometry. Clinical Microbiology and Infection, 17, 1735–1739. 10.1111/j.1469-0691.2011.03468.x 21375659

[mbo3784-bib-0009] Carlton, R. M. , Noordman, W. H. , Biswas, B. , de Meester, E. D. , & Loessner, M. J. (2005). Bacteriophage P100 for control of *Listeria monocytogenes* in foods: Genome sequence, bioinformatic analyses, oral toxicity study, and application. Regulatory Toxicology and Pharmacology, 43, 301–312. 10.1016/j.yrtph.2005.08.005 16188359

[mbo3784-bib-0010] Carvalho, C. M. , Gannon, B. W. , Halfhide, D. E. , Santos, S. B. , Hayes, C. M. , Roe, J. M. , & Azeredo, J. (2010). The in vivo efficacy of two administration routes of a phage cocktail to reduce numbers of *Campylobacter coli* and *Campylobacter jejuni* in chickens. BMC Microbiology, 10, 232 10.1186/1471-2180-10-232 20809975 PMC2940857

[mbo3784-bib-0011] Carvalho, C. M. , Susano, M. A. , Fernandes, E. , Santos, S. B. , Gannon, B. W. , Nicolau, A. , … Azeredo, J. (2010). Method for bacteriophage isolation against target *Campylobacter* strains. Letters in Applied Microbiology, 50, 192–197. 20002571 10.1111/j.1472-765X.2009.02774.x

[mbo3784-bib-0012] Coles, F. M. , McCarthy, N. D. , Bliss, C. M. , Layton, R. , & Maiden, M. C. (2015). The long‐term dynamics of *Campylobacter *colonizing a free‐range broiler breeder flock: An observational study. Environmental Microbiology, 17, 938–946. 25588789 10.1111/1462-2920.12415 PMC4390391

[mbo3784-bib-0013] Dec, M. , Nowaczek, A. , Urban‐Chmiel, R. , Stępień‐Pyśniak, D. , & Wernicki, A. (2018). Probiotic potential of *Lactobacillus* isolates of chicken origin with anti‐Campylobacter activity. The Journal of Veterinary Medical Science, 80, 1195–1203. 29877314 10.1292/jvms.18-0092 PMC6115247

[mbo3784-bib-0014] Dudzic, A. , Urban‐Chmiel, R. , Stępień‐Pyśniak, D. , Dec, M. , Puchalski, A. , & Wernicki, A. (2016). Isolation, identification and antibiotic resistance of *Campylobacter *strains isolated from domestic and free‐living pigeons. British Poultry Sciences, 57, 172–178. 10.1080/00071668.2016.1148262 26841300

[mbo3784-bib-0015] EFSA (European Food Safety Authority) and ECDC (European Centre for Disease Prevention and Control) . (2014). The European Union summary report on trends and sources of zoonoses, zoonotic agents and food-borne outbreaks in 2012. EFSA Journal, 12(2), 3590, 312 p. 10.2903/j.efsa.2014.3547 PMC7009540 32625785

[mbo3784-bib-0016] EFSA and ECDC . (2015). The European Union summary report on trends and sources of zoonoses, zoonotic agents and food‐borne outbreaks in 2014. EFSA Journal, 13, 3911 10.2903/j.efsa.2015.4329. PMC7009540 32625785

[mbo3784-bib-0017] EFSA and ECDC . (2016). The European Union summary report on antimicrobial resistance in zoonotic and indicator bacteria from humans, animals and food in the European Union in 2014. EFSA Journal, 14, 4380 10.2903/j.efsa.2016.4380. PMC13093165 42016873

[mbo3784-bib-0018] EUCAST, European Committee on Antimicrobial Susceptibility Testing . (2016a). *Antimicrobial wild type distribution of microorganisms. C. jejuni* .

[mbo3784-bib-0019] EUCAST, European Committee on Antimicrobial Susceptibility Testing . (2016b). *Antimicrobial wild type distribution of microorganisms. C. coli* .

[mbo3784-bib-0020] Firlieyanti, A. S. , Connerton, P. L. , & Connerton, I. F. (2016). Campylobacters and their bacteriophages from chicken liver. The prospect for phage biocontrol. International Journal of Food Microbiology, 237, 121–127. 27565524 10.1016/j.ijfoodmicro.2016.08.026 PMC5064024

[mbo3784-bib-0021] Furuta, M. , Nasu, T. , Umeki, K. , Minh, D. F. , Honjoh, K.‐I. , & Miyamoto, T. (2017). Characterization and application of lytic bacteriophages against *Campylobacter jejuni* isolated in poultry in Japan. Biocontrol Science., 22, 213–221. 29279578 10.4265/bio.22.213

[mbo3784-bib-0022] Hagens, S. , & Loessner, M. (2010). Bacteriophage for biocontrol of foodborne pathogens: Calculations and considerations. Current Pharmaceutical Biotechnology, 11, 58–68. 20214608 10.2174/138920110790725429

[mbo3784-bib-0023] Han, J. E. , Kim, J. H. , Hwang, S. Y. , Choresca, C. H. Jr , Shin, S. P. , Jun, J. W. , … Park, S. C. (2013). Isolation and characterization of a *Myoviridae* bacteriophage against *Staphylococcus aureus* isolated from dairy cows with mastitis. Research in Veterinary Sciences, 95, 758–763. 10.1016/j.rvsc.2013.06.001 23790669

[mbo3784-bib-0024] Hansen, V. M. , Rosenquist, H. , Baggesen, D. L. , Brown, S. , & Christensen, B. B. (2007). Characterization of *Campylobacter *phages including analysis of host range by selected *Campylobacter *Penner serotypes. BMC Microbiology, 7, 90 10.1186/1471-2180-7-90 17945022 PMC2194700

[mbo3784-bib-0025] Hwang, S. , Yun, J. , Kim, K. P. , Heu, S. , Lee, S. , & Ryu, S. (2009). Isolation and characterization of bacteriophages specific for *Campylobacter jejuni* . Microbiology and Immunology, 53, 559–566. 19780969 10.1111/j.1348-0421.2009.00163.x

[mbo3784-bib-0026] Ingresa‐Capaccioni, S. , González‐Bodí, S. , Jiménez‐Trigos, E. , Marco‐Jiménez, F. , Catalá, P. , Vega, S. , & Marin, C. (2015). Comparison of different sampling types across the rearing period in broiler flocks for isolation of *Campylobacter *spp. Poultry Sciences, 94, 766–771. 10.3382/ps/pev023 25743419

[mbo3784-bib-0027] Lemos, A. , Morais, L. , Fontes, M. , Pires, I. , & Vieira‐Pinto, M. (2015). *Campylobacter* spp. isolation from infected poultry livers with and without necrotic lesions. Food Control, 50, 236–242. 10.1016/j.foodcont.2014.08.027

[mbo3784-bib-0028] Lim, T. H. , Lee, D. H. , Lee, Y. N. , Park, J. K. , Youn, H. N. , Kim, M. S. , … Song, C. S. (2011). Efficacy of bacteriophage therapy on horizontal transmission of *Salmonella *Gallinarum on commercial layer chickens. Avian Diseases, 55, 435–438. 10.1637/9599-111210-Reg.1 22017042

[mbo3784-bib-0029] Loc Carrillo, C. , Atterbury, R. J. , Connerton, P. L. , Dillon, E. , Scott, A. , & Connerton, I. F. (2005). Bacteriophage therapy to reduce *Campylobacter jejuni *colonization of broiler chickens. Applied Environmental Microbiology, 71, 6554–6563. 10.1128/AEM.71.11.6554-6563.2005 16269681 PMC1287621

[mbo3784-bib-0030] Loc Carrillo, C. M. , Connerton, P. L. , Pearson, T. , & Connerton, I. F. (2007). Free‐range layer chickens as a source of *Campylobacter* bacteriophage. Antonie Van Leeuwenhoek, 92, 275–284. 10.1007/s10482-007-9156-4 17387630

[mbo3784-bib-0031] Maron, D. F. , Smith, T. J. S. , & Nachman, K. E. (2013). Restrictions on antimicrobial use in food animal production: An international regulatory and economic survey. Global Health, 9, 48 10.1186/1744-8603-9-48 24131666 PMC3853314

[mbo3784-bib-0032] Martiny, D. , Visscher, A. , Catry, B. , Chatellier, S. , & Vandenberg, O. (2013). Optimization of *Campylobacter* growth conditions for further identification by matrix assisted laser desorption/ionization time‐of‐flight mass spectrometry (MALDI‐TOF MS). Journal Microbiol Methods, 94, 221–223. 10.1016/j.mimet.2013.06.018 23811211

[mbo3784-bib-0033] O’Flaherty, S. , Ross, R. P. , & Coffey, A. (2009). Bacteriophage and their lysins for elimination of infectious bacteria. FEMS Microbiology Reviews, 33, 801–819. 10.1111/j.1574-6976.2009.00176.x 19416364

[mbo3784-bib-0034] Owens, J. , Barton, M. D. , & Heuzenroeder, M. W. (2013). The isolation and characterization of *Campylobacter jejuni* bacteriophages from free range and indoor poultry. Veterinary Microbiology, 162, 144–150. 10.1016/j.vetmic.2012.08.017 22980913

[mbo3784-bib-0035] Prachantasena, S. , Charununtakorn, P. , Muangnoicharoen, S. , Hankla, L. , Techawal, N. , Chaveerach, P. , … Luangtongkum, T. (2016). Distribution and genetic profiles of *Campylobacter* in commercial broiler production from breeder to slaughter in Thailand. PLoS ONE, 1(2), e0149585 10.1371/journal.pone.0149585 PMC4757449 26886590

[mbo3784-bib-0036] Sails, A. D. , Wareing, D. R. , Bolton, F. J. , Fox, A. J. , & Curry, A. (1998). Characterization of 16 *Campylobacter jejuni *and *C. coli *typing bacteriophages. Journal of Medical Microbiology, 47, 123–128. 9879954 10.1099/00222615-47-2-123

[mbo3784-bib-0037] Sørensen, M. C. H. , Gencay, Y. E. , Birk, T. , Baldvinsson, S. B. , Jäckel, C. , Hammerl, J. A. , … Brøndsted, L. (2015). Primary isolation strain determines both phage type and receptors recognised by *Campylobacter jejuni* bacteriophages. PLoS ONE, 10(1), e0116287– 10.1371/journal.pone.0116287 25585385 PMC4293142

[mbo3784-bib-0038] Stępień‐Pyśniak, D. , Hauschild, T. , Różański, P. , & Marek, A. (2017). MALDI‐TOF mass spectrometry as a useful tool for identification of *Enterococcus *spp. from wild birds and differentiation of closely related species. Journal of Microbiology and Biotechnology, 27, 1128–1137. 28285496 10.4014/jmb.1612.12036

[mbo3784-bib-0039] Weber‐Dąbrowska, B. , Mulczyk, M. , & Górski, A. (2000). Bacteriophage therapy of bacterial infections: An update of our institute’s experience. Archivum Immunologiae Et Therapiae Experimentalis, 48, 547–551. 11197610

[mbo3784-bib-0040] Weinbauer, M. G. (2004). Ecology of prokaryotic viruses. FEMS Microbiology Reviews, 28, 127–181. 10.1016/j.femsre.2003.08.001 15109783

[mbo3784-bib-0041] Wernicki, A. , Nowaczek, A. , & Urban‐Chmiel, R. (2017). Bacteriophage therapy to combat bacterial infections in poultry. Virology Journal, 14, 179 10.1186/s12985-017-0849-7 28915819 PMC5602926

[mbo3784-bib-0042] Wieczorek, K. , & Osek, J. (2015). A five‐year study on prevalence and antimicrobial resistance of *Campylobacter* from poultry carcasses in Poland. Food Microbiology, 49, 161–165. 25846926 10.1016/j.fm.2015.02.006

[mbo3784-bib-0043] Wieczorek, K. , Szewczyk, R. , & Osek, J. (2012). Prevalence, antimicrobial resistance and molecular characterization of *Campylobacter jejuni* and *C. coli* isolated from retail raw meat in Poland. Veterinarni Medicina, 57, 293–299. 10.17221/6016-VETMED

[mbo3784-bib-0044] Woźniak, A. , & Wieliczko, A. (2011). Tetracycline, erythromycin, and gentamicin resistance of *Campylobacter jejuni* and *Campylobacter coli* isolated from poultry in Poland. Bulletin of Veterinary Institute in Pulawy, 55, 51–54.

