# Supplementary material for: Campylobacter spp. and bacteriophages from broiler chickens: Characterization of antibiotic susceptibility profiles and lytic bacteriophages
Source: Microbiologyopen. 2019 Jan 17;8(7):e00784. doi: 10.1002/mbo3.784 (PMC6612548; doi:10.1002/mbo3.784)
Supplement: Supplementary file 1 [file MBO3-8-e00784-s001.docx]

| **Antomicrobial resistance profiles** | **Number of resistant strains with the profile** | **Number of resistant isolates** | |
| --- | --- | --- | --- |
|  |  | ***C. jejuni*** | ***C. coli*** |
| **CIP, T, CN, S, E, AMP, AML, C** | 2 | 1 | 1 |
| **CIP, T, CN, E, AMP, AML, C** | 3 |  | 3 |
| **CIP, T, CN, S, E , AMP, AML** | 2 | 1 | 1 |
| **CIP, T, CN, E, AMP, AML** | 1 |  | 1 |
| **CIP, T, CN, S, E, AMP** | 1 | 1 |  |
| **CIP, CN, S, E, AMP, AML** | 1 | 1 |  |
| **CIP, CN, E, AMP, AML** | 2 | 2 |  |
| **CIP, T, CN, AMP, AML** | 1 |  | 1 |
| **CIP, T, E, AMP** | 1 |  | 1 |
| **CIP, S, E, AMP** | 1 | 1 |  |
| **CIP, T, CN, E** | 2 | 1 | 1 |
| **CIP, T, S, C** | 1 | 1 |  |
| **CIP, CN, AMP, AML** | 3 | 3 |  |
| **CIP, E, AMP, AML** | 2 | 1 | 1 |
| **CIP, T, AMP** | 1 |  | 1 |
| **CIP, E, AMP** | 1 | 1 |  |
| **CIP, T, CN** | 5 | 3 | 2 |
| **CIP, T, S** | 3 | 2 | 1 |
| **CIP, T, E** | 5 | 4 | 1 |
| **CIP, CN, E** | 2 | 2 |  |
| **T, CN, C** | 2 | 1 | 1 |
| **T, S, C** | 1 | 1 |  |
| **T, E, C** | 1 |  | 1 |
| **T, S** | 2 | 2 |  |
| **T** | 2 | 2 |  |

Table. 3 a. The antibiotic profiles among the tested *C. jejuni* and *C. coli* strains

Legend: AML, AMP- β-lactams; CIP-fluoroquinolon, CN,S- amonoglycosides, T- tetracycline,

E- macrolide, C- miscellaneous.
